# Successful therapy of a newborn with *Stenotrophomonas maltophilia* nosocomial pneumonia with cefiderocol

**DOI:** 10.1007/s15010-024-02404-9

**Published:** 2024-10-07

**Authors:** Janina Trauth, Rahel Schuler, Markus Waitz, Harald Ehrhardt, Moritz Fritzenwanker, Susanne Herold

**Affiliations:** 1 Department of Medicine V - Internal Medicine, Infectious Diseases & Infection Control, Justus- Liebig-University, member of the German Center for Lung Research (DZL) and German Center for Infection Research (DZIF) and Cardio-Pulmonary Institute (CPI), Giessen, Germany; 2 Department of General Pediatrics and Neonatology, Justus Liebig University, Giessen, Germany; 3 Institute of Medical Microbiology, Justus Liebig University Giessen, Giessen, Germany; 4 Division of Neonatology and Pediatric Intensive Care Medicine, Department of Pediatrics and Adolescent Medicine, University Medical Center Ulm, Ulm, Germany

**Keywords:** Multi drug resistance, *Stenotrophomonas maltophilia*, Cefiderocol, Pneumogenic sepsis, Neonate

## Abstract

Cefiderocol is a new siderophore-beta-lactam antibiotic used for the treatment of severe multidrug-resistant infections like sepsis, hospital-acquired and ventilator-associated pneumonia in adults, but there are only single reports on its use in the neonatal population. We describe the successful cefiderocol treatment of a newborn with pneumogenic sepsis due to *Stenotrophomonas maltophilia*.

## Case presentation

*Stenotrophomonas maltophilia*, a Gram-negative aerobic nonfermenter bacterium, is a ubiquitous, opportunistic pathogen that is commonly found in the environment. Although *S. maltophilia* infections are concerning due to several intrinsic resistance mechanisms, they are relatively uncommon compared to other microorganisms. *S. maltophilia* infections mainly affect patients with risk factors like immunocompromised, oncologic or other patients at risk of infection, patients under broad-spectrum antibiotic therapy and patients with artificial ventilation or central venous catheters. In pediatric and neonatal units, multidrug-resistant (MDR) Gram-negative bacteria like *S. maltophilia* are an increasingly significant cause of morbidity and mortality [1–3]. Infections manifest themselves as sepsis, pneumonia, meningitis, endophthalmitis, endocarditis, wound infections, and urinary tract infections [4]. The respiratory tract has been found to be the most frequent site of infection [5]. Many infections are polymicrobial, e.g. occurring with other nonfermenters like *Pseudomonas aeruginosa*. Most treatment options for *P. aeruginosa* are not recommended for *S. maltophilia* infections due to natural resistance to most antibiotics. This resistance allows *S. maltophilia*, in settings where carbapenems are used extensively, to colonise patients with dysbiosis, and potentially turning colonisation into invasive infection. Mortality rates of infections can be up to 70%. Treatment options are limited to reserve antibiotics such as cefiderocol [5–7, 8].

Cefiderocol is a novel siderophore cephalosporin antibiotic. Siderophores are low molecular weight iron-chelating compounds synthesized by microbial pathogens and secreted under iron-limited conditions. Cefiderocol carries a catechol moiety on the 3-position side chain, which forms a chelating complex with ferric iron. This induces active uptake of the antibiotic-iron-complex by iron transport system of Gram negative bacteria (including *S. maltophilia*), thus enhancing the concentration of the compound at the desired locus of operation, the bacterial cell wall. This yields very low minimal inhibitory concentrations (MIC) in vitro (even with strains that are otherwise highly resistant to beta-lactam antibiotics), which suggests advantageous opportunities for clinical therapy of infections with multiresistant Gram negative rods [9, 10]. It is used for the treatment of severe infections like sepsis, hospital-acquired (HAP) and ventilator-associated pneumonia (VAP) in adults [11] but there are only very few data on its use in neonates [1, 11–14]. Cefiderocol has demonstrated excellent in vitro efficacy against *S. maltophilia*, including strains resistant to other recommended antibiotics like TMP/SMX [5, 15].

We report a case of a female preterm infant, delivered via secondary emergency caesarean section at 36 + 2 weeks gestation for pathological cardiotocography (CTG). Postnatally, the patient was treated on NICU (neonatal intensive care unit) due to left-sided congenital diaphragmatic hernia (CDH), intrauterine growth restriction (IUGR) and a birth weight of 2000 g. Later, it was found to have a mosaic trisomy 13.

**Fig. 1:**
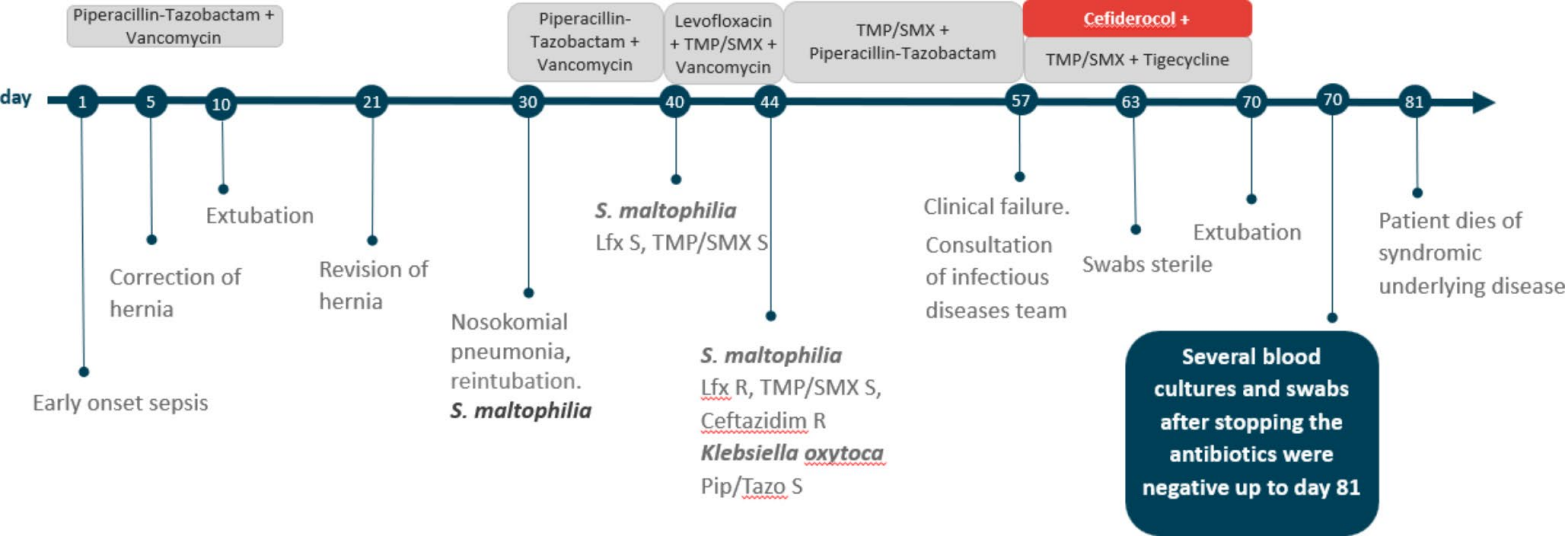
Longitudinal course with details on the microbial colonization, infections, and antibiotic therapy. Pip/Tazo: piperacillin-tazobactam, TMP/SMX: trimethoprim-sulfamethoxazole, Lfx: levofloxacin, S: sensitive. R: resistant

As early-onset infection was suspected, empiric antibiotic treatment with piperacillin-tazobactam (80 mg/kg qid) and vancomycin (20 mg/kg tid) was given 14 days, according to the patient’s risk of ESBL and MRSA carriage (Fig. 1). Correction of the CDH was performed on day 5. After successful extubation on day 10 and hernia revision surgery on day 21, nosocomial pneumonia was diagnosed on day 30, which required reintubation and mechanical ventilation. No other focus was found, and blood cultures were negative. Empirical treatment with piperacillin-tazobactam and vancomycin was again initiated leading to clinical improvement and decline of CRP. *S. maltophilia* was found in tracheal aspirates, but initially thought to be a coloniser. The isolate was cultivated on Columbia Sheep Blood Agar (Thermo Scientific, USA) and MacConkey Agar (Oxoid Ltd., UK), the species was determined by mass spectrometry on the Vitek System, susceptibility testing was initially done on the Vitek 2 system, using the AST-N263 GNS card (Biomerieux, France).

On day 40, the child presented again with fever and higher oxygen demand. Microbiological results of tracheal aspirates still showed only *S. maltophilia*. The isolate was cultivated the same way as before, resistance testing was expanded by using etests (epsilometer tests) (Liofilchem, Italy) for levofloxacin and trimethoprim-sulfamethoxazole on Mueller-Hinton-Agar (Oxoid Ltd, UK), McFarland 0.5, according to EUCAST standards.

Therapy was changed to levofloxacin 10 mg/kg bid in combination with TMP/SMX 5 mg/kg bid (TMP). By day 44, CRP did not adequately decline, and *S. maltophilia* now showed new resistance to levofloxacin (MIC 32 mg/l) and ceftazidime (MIC 256 mg/l) (epsilometer tests as above). Further microbiological susceptibility testing showed *S. maltophilia* susceptibility against cefiderocol (disk diffusion test on Mueller-Hinton-Agar, McFarland 0.5, 30 µg cefiderocol disk - the zone diameter was measured as 29 mm – diameters of at least 20 mm correspond to MIC value below 2 mg/dl, according to EUCAST). Additional susceptibility tests with epsilometer tests were done for TMP/SMX (MIC 1.5 mg/l), and tigecycline (MIC 0.19 mg/l, no cutoff for interpretation), on Mueller-Hinton agar, McFarland 0.5. A second pathogen was detected in tracheal aspirate, *Klebsiella oxytoca (K. oxytoca)*, susceptible to piperacillin-tazobactam, TMP/SMX and levofloxacin. Treatment was therefore extended and piperacillin-tazobactam was added. After an initial clinical improvement and declining CRP, the patient deteriorated on day 57. After interdisciplinary discussion including the infectious diseases team, the antibiotic regimen was changed to cefiderocol 100 mg/kg tid plus dose-escalated TMP/SMX 12 mg/kg bid plus tigecycline 1,5 mg/kg bid. After 5 days nasal, oropharyngeal and anal swabs and tracheal aspirates remained sterile. The total 2-week treatment with cefiderocol plus TMP/SMX and tigecycline led to clinical and microbiological cure. On day 70, the baby was successfully extubated, and all antibiotic therapy was stopped. No toxicities under the combination treatment were observed. Several blood cultures and all swabs after stopping the antibiotics were negative up to day 81, and the patient had no further clinical or serological signs of infection (CRP < 0,5 mg/l). There were no other children in the ward at any time colonized or infected with this *S. maltophilia* strain.

Nevertheless, the patient’s severe birth defect (CDH) with severe pulmonary hypoplasia and pulmonary hypertension (PHTN) resulted in chronic respiratory insufficiency and permanent dependency on continuous positive airway pressure (CPAP). Therefore, together with the parents, the consensual decision for palliative care was made. The patient died of her underlying syndromic disease on day 81.

## Discussion

Severe infections in neonates, like sepsis or nosocomial pneumonia including VAP, are great challenges for paediatric physicians, especially if these infections are caused by multi-drug resistant Gram-negative bacteria. *S. maltophilia* is a ubiquitous, opportunistic pathogen mostly causing hospital-associated infections and particularly associated with the previous use of broad spectrum beta lactams. Clinicians often face the difficulty to determine true infection with *S. maltophilia* versus colonisation (for example colonisation of the airways and tracheal tube). Using broad spectrum antibiotics in critically ill patients, e.g. on NICU, promote subsequent *S. maltophilia* colonisation and then infection. Our female neonate empirically treated with piperacillin-tazobactam plus vancomycin for suspected early-onset infection later acquired pneumonia due to *S. maltophilia* and *K. oxytoca*. *S. maltophilia* is a known cause of VAP in neonates with high morbidity and mortality risk due to intrinsic resistance to multiple classes of antibiotics, including carbapenems [2]. An L1 MBL and L2 serine β-lactamase render most conventional β-lactams ineffective against *S. maltophilia.* L1 hydrolyzes penicillins, cephalosporins, and carbapenems, but not aztreonam. L2 hydrolyzes extended-spectrum cephalosporins and aztreonam. Current IDSA guidelines recommend the use of two of the following agents for *S. maltophilia*: cefiderocol, minocycline, TMP/SMX, or levofloxacin, or the combination of ceftazidime-avibactam and aztreonam. Cefiderocol as a component of combination therapy, at least until clinical improvement is observed, is a preferred agent. Aminoglycosides are commonly used in NICUs, especially in the context of Gram negative sepsis (whilst awaiting final culture results), but are no appropriate therapy for *S. maltophilia*: the pathogen can accumulate multidrug efflux pumps and chromosomal resistance genes for aminoglycoside inactivating enzymes (aminoglycoside acetyl transferase enzyme). TMP/SMX was a remaining option in our case; however, rising rates of TMP/SMX resistance in *S. maltophilia* isolates due to multidrug efflux pumps have been reported [16]. Furthermore, TMP/SMX can cause hyperbilirubinemia and bone marrow suppression, and only limited data is published on appropriate dosing in neonates. In our case, development of levofloxacin- and ceftazidime-resistance during antibiotic therapy further reduced the treatment options. Our patient was treated with six different antibiotics against Gram-negative bacteria, and until now, none of them is approved for neonates. Switching to cefiderocol plus TMP/SMX plus tigecycline led to sterile sputa after only 5 days. The total 2-week treatment with this combination therapy led to a clinical and microbiological. However, as TMP/SMX dose-escalation, tigecycline and cefiderocol therapy were started all simultaneously, we cannot determine which substance or combination led to cure. Toxicities were not observed for the used cefiderocol dose of 100 mg/kg tid.

Several reports of cefiderocol used in a dose of 30 mg/kg tid to treat MDR *P. aeruginosa*, *K. pneumoniae* and *Achromobacter xylosoxidans* in children and neonates can be found in the literature [1, 12, 13, 17–20]. Experimental data in vitro and in vivo point to a dose-dependent effect up to 100 mg/kg, and dosing for adults for cefiderocol is mostly 60-100 mg/kg, so 100 mg/kg was chosen for our patient. The substance shows high stability against metallo-β-lactamase producing Gram-negative pathogens causing neonatal morbidity and mortality worldwide [21]. There is a low risk for drug-drug interactions, a special advantage in the neonatal intensive care setting, where different drugs due to potential comorbidities may be necessary. Iron hemostasis remains unaffected [10]. The favourable safety profile of the cephalosporins that has been proven for cefiderocol in adults with severe infections in clinical trials and under real world conditions however needs to be proven in infants. Three clinical studies are currently recruiting to assess pediatric safety, pharmacokinetics, and tolerability of cefiderocol 60 mg/kg tid in neonates and infants less < 3 months of age (NCT06086626), > 3 months (NCT04335539), and in children aged 3 months to less than 18 years (NCT04215991).

In many cases, off-label antibiotic use in children, and especially in neonates, is life-saving in pediatric intensive care units [22]. Our experience of well-tolerated cefiderocol in a high-dose of 100 mg/kg/d in a preterm neonate with low birth weight underlines the favourable profile and clinical efficacy of this substance in neonates. Still, this is one case report only and further safety and dosing data for cefiderocol are required. However, this is extremely promising and, whilst better data are not available, is an important case for clinicians to be aware of.

## Data Availability

No datasets were generated or analysed during the current study.
