# Correction: Successful therapy of a newborn with *Stenotrophomonas maltophilia* nosocomial pneumonia with cefiderocol

**DOI:** 10.1007/s15010-025-02487-y

**Published:** 2025-02-24

**Authors:** Janina Trauth, Rahel Schuler, Markus Waitz, Harald Ehrhardt, Moritz Fritzenwanker, Susanne Herold

**Affiliations:** 1 Department of Medicine V - Internal Medicine, Infectious Diseases & Infection Control, Member of the German Center for Lung Research (DZL) and German Center for Infection Research (DZIF) and Cardio-Pulmonary Institute (CPI), Justus- Liebig-University, Giessen, Germany; 2 Department of General Pediatrics and Neonatology, Justus Liebig University, Giessen, Germany; 3 Division of Neonatology and Pediatric Intensive Care Medicine, Department of Pediatrics and Adolescent Medicine, University Medical Center Ulm, Ulm, Germany; 4 Institute of Medical Microbiology, Justus Liebig University Giessen, Giessen, Germany

**Infection (2024)**

10.1007/s15010-024-02404-9

The affiliation of

Janina Trauth

janina.trauth@innere.med.uni-giessen.de

Department of Medicine V - Internal Medicine, Infectious Diseases & Infection Control, Justus- Liebig-University,Giessen, Germany has been published incomplete.

Correct is:

Janina Trauth

janina.trauth@innere.med.uni-giessen.de

Department of Medicine V - Internal Medicine, Infectious Diseases & Infection Control, Justus- Liebig-University, Giessen, Germany, member of the German Center for Lung Research (DZL) and German Center for Infection Research (DZIF) and Cardio-Pulmonary Institute (CPI)

The Original Article has been corrected.

